# Dynamic Liver Function Tests in Paediatric Liver Disease

**DOI:** 10.3390/diagnostics16050805

**Published:** 2026-03-09

**Authors:** Thora Wesenberg Helt, Jon Nielsen, Gabriella Ficerai-Garland, Robin de Nijs, Christina Louise Winther, Søren Møller, Viktoria Setterberg, Vibeke Brix Christensen, Lise Borgwardt

**Affiliations:** 1 Department of Clinical Physiology and Nuclear Medicine, Copenhagen University Hospital—Rigshospitalet, 2100 Copenhagen, Denmark; 2 Department of Paediatrics and Adolescent Medicine, Copenhagen University Hospital—Rigshospitalet, 2100 Copenhagen, Denmark; 3 School of Medicine, University of Pittsburgh, Pittsburgh, PA 15260, USA; ficerai-garland.gabriella@medstudent.pitt.edu; 4 Department of Clinical Physiology and Nuclear Medicine, Copenhagen University Hospital—Hvidovre, 2650 Hvidovre, Denmark; 5 Department of Clinical Medicine, Faculty of Health Sciences, University of Copenhagen, 2200 Copenhagen, Denmark; 6 Department of Pediatric Surgery, Copenhagen University Hospital—Rigshospitalet, 2100 Copenhagen, Denmark; 7 Department of Comparative Pediatrics, University of Copenhagen, 1870 Copenhagen, Denmark

**Keywords:** paediatric liver disease, indocyanine green clearance, hepatobiliary scintigraphy, liver function

## Abstract

**Background/Objectives**: Liver function is difficult to estimate accurately. Conventional liver function tests can be normal, even in severe diseases. Dynamic liver function tests, including indocyanine green (ICG) clearance and hepatobiliary scintigraphy (HBS), are useful in adults. We aimed to evaluate the association between ICG clearance and HBS in children with liver disease and to identify liver disease markers associated with liver function measured with ICG clearance and HBS. **Methods**: Children aged 0–18 years followed at Copenhagen University Hospital, Rigshospitalet between November 2015 and August 2024 were eligible for inclusion if they had acute or chronic liver disease, suspected liver disease, or previous liver transplantation (LTx). All underwent ICG clearance and HBS. **Results**: We included 131 children with a total of 200 visits. The median visit age was 11.4 [6.6; 15.6] years. The ICG-plasma disappearance rate had the strongest correlation with the hepatic extraction fraction (ρ = 0.64, *p* < 0.001). ICG clearance and HBS were associated with liver injury, reduced synthetic function, cholestasis, cirrhosis, and portal hypertension, while only ICG clearance was associated with the portal blood flow. LTx was associated with increased HBS parameters, but not with ICG clearance. **Conclusions**: ICG clearance and HBS are correlated, and both are associated with most conventional liver function markers. This suggests their usefulness in evaluating children with liver disease. However, further evaluation of the predictive and clinical value of ICG clearance and HBS in disease progression is needed.

## 1. Introduction

Paediatric liver disease (PLD) has significant morbidity and mortality, with over 15,000 children hospitalised annually in the United States [1,2,3]. PLD includes a wide range of aetiologies with varying severity from mild to fatal disease, highlighting the need to accurately predict the prognosis and timing of treatment [1]. Yet, the liver has many functions, including macronutrient metabolism and storage, coagulation, and the breakdown and excretion of drugs, making the assessment of liver function challenging [4,5]. Due to the multifunctional role of the liver, no single test can provide an accurate estimate of all liver functions. While liver biopsy is the gold standard for assessing liver fibrosis, it is invasive, carries a risk of infection and bleeding, and requires general anaesthesia and hospitalisation in children. It also provides limited information on the function of non-fibrotic liver cells [6,7]. Conventional liver function tests from readily available blood tests provide an indirect measurement of overall liver function by assessing liver injury, synthetic function, and cholestasis [5]. However, these tests vary with acute infection and vitamin K deficiency and can be within a normal range, even in patients with cirrhosis, limiting their clinical value [5,8].

In contrast, dynamic liver function tests such as indocyanine green (ICG) clearance and hepatobiliary scintigraphy (HBS) provide information about the metabolic function of hepatocytes, with both decreased in cirrhosis [5,9]. ICG is almost exclusively extracted by hepatocytes and is excreted unchanged into the bile [10]. The remaining ICG in the blood can be measured non-invasively via pulse spectrophotometry. HBS uses the 99mTc-labelled analogue of iminodiacetic acid ([^99m^Tc]-IDA), which follows the excretory pathway of bilirubin [5]. Both modalities are useful to determine liver function in adults and are highly correlated [11,12]. However, few studies have evaluated the use of ICG clearance and HBS in children and their association with other liver function tests. No studies have evaluated the association between ICG clearance and HBS [13,14], which is important, as ICG clearance primarily reflects cellular uptake and HBS reflects uptake and excretion.

We hypothesised that both modalities would be useful for assessing liver function in children, as each are associated with key markers of disease severity. We further hypothesised that ICG clearance and HBS would show a strong correlation. This study aimed to evaluate their association in children with liver disease and to identify conventional disease markers linked to liver function, as measured by these methods.

## 2. Materials and Methods

### 2.1. Study Design, Setting, and Participants

This prospective cohort study included children with liver disease aged 0–18 years, who were followed at the Department of Paediatrics and Adolescent Medicine and Department of Paediatric Surgery, Copenhagen University Hospital—Rigshospitalet. All eligible children from November 2015 to August 2024 were invited to participate. Patients were eligible if they had acute or chronic liver disease, had suspected liver disease (elevated liver enzymes) or a previous liver transplantation (LTx). Patients were excluded if they were unfit for blood sampling (e.g., due to clinical condition, size, age, or respiratory compromise), had an iodine allergy or thyrotoxicosis due to the iodine content in ICG, or if they were unfit for hepatobiliary scintigraphy (e.g., due to a rare allergic reaction to the tracer or relative contraindications related to patient preparation). This study was part of a prospective cohort study, where the children were followed up after 6 to 12 months, 3 years and 5 years. Measurements from these visits were included if ICG clearance and HBS were within seven days of each other for stable liver disease and within one day of each other for acute liver disease (INR ≥ 1.5 or toxic liver disease). Written informed consent was obtained from all custody holders and from participants aged 18 years. This study was performed in line with the principles of the Declaration of Helsinki. The study was approved by the National Committee on Health Research Ethics of Denmark (H-15009740, 8 October 2015). The study was registered at ClinicalTrials.gov (NCT03509194) on 17 December 2017.

### 2.2. Data Collection

Age was calculated as the difference between birth and the date of HBS. Acute liver disease was defined as INR ≥ 1.5 or toxic liver disease with no known chronic liver disease. LTx was defined as liver transplantation performed before both dynamic liver function tests (HBS and ICG clearance). Blood tests (alanine aminotransferase (ALT) (U/L), gamma-glutamyl transferase (GGT) (U/L), total and conjugated bilirubin (µM), international normalised ratio (INR), prothrombin–proconvertin clotting time (PP), platelets (10^9^/L), and albumin (g/L)) were measured within seven days from both HBS and ICG clearance if clinically stable or if unstable within one day. They were collected from LABKA II (Dedalus Healthcare Denmark ApS, Aarhus, Denmark). The portal blood flow was obtained from their clinical liver ultrasound in Sectra IDS7 (Sectra AB, Linköping, Sweden). The portal blood flow was included if the ultrasound was performed within 3 months of HBS or if the ultrasound was performed both before and after HBS with the same result. Fibroscan (Echosens, Paris, France) was performed within 3 months of HBS, as previously described [15]; if that was not possible, Fibroscan was accepted if they had stable liver disease and had two scans with similar results before and after HBS. The fibrosis stage was collected for those who clinically required a liver biopsy, as previously described [15]. The METAVIR scoring system was used to assess fibrosis on a scale of 0–4, as follows: F0, no fibrosis; F1, mild fibrosis, portal fibrosis without septa; F2, significant fibrosis, portal fibrosis with rare septa; F3, severe fibrosis, numerous septa without cirrhosis; and F4, cirrhosis. Total bilirubin was categorised as in >20 µM or ≤20 µM for the analysis of cholestasis. Portal blood flow was categorised as <20 cm/s or ≥20 cm/s. Patients with a transjugular intrahepatic portosystemic shunt were defined as having portal blood flow < 20 cm/s. Diseases were grouped as metabolic/genetic, biliary atresia, autoimmune hepatitis, or other (Table 1).

### 2.3. Hepatobiliary Scintigraphy

All hepatobiliary scans were performed at Copenhagen University Hospital—Rigshospitalet. A total of 20–120 [^99m^Tc]-mebrofenin was administered intravenously according to body weight, using an EANM dosing card [16] with a minimum activity of 20 MBq for infants 0–8 kg. The camera was a gamma camera (Siemens SPECT/CT Symbia T16 or Siemens SPECT Evo, Siemens Healthineers, Erlangen, Germany) equipped with low-energy high-resolution parallel hole collimators. The energy window for ^99m^Tc was set at 140 keV, with 15% width. The matrix size was 128 × 128 and zoom was between 1.45 and 2.29, resulting in an isotropic pixel size of 3.3 mm for zoom of 1.45 and 2.1 mm for zoom of 2.29. Images were obtained for one hour in the anterior view (1 s frames for the first minute, 10 s frames for the next 3 min, and 60 s frames for the last 56 min, constituting 134 frames in total) to visualise the liver, bile ducts, and gallbladder [13,17]. Subjects fasted for two hours and scanned in the supine position. Distraction techniques and appropriate immobilisation was used to avoid patient movement and upper limb injections, preferably in the antecubital fossa, were performed with a fast intact bolus to optimise the deconvolution analysis technique.

**Figure 1:**
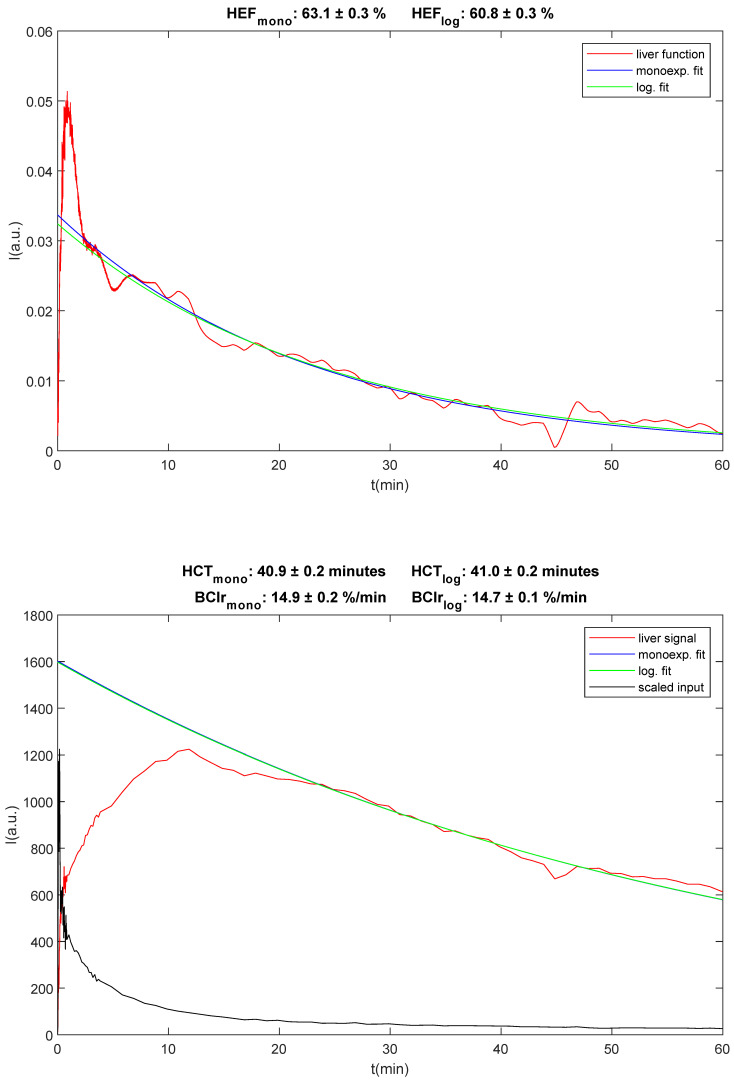
An example of calculation of hepatic extraction fraction (HEF), hepatic clearance time (HCT), and blood clearance (BClr).

HBSs were analysed and quantitative measures of hepatic clearance, hepatic extraction fraction (HEF, %), blood clearance (BClr, %/min) and the hepatic clearance rate (HCR, %/min) were calculated. HEF and hepatic clearance time (HCT) were calculated using the method previously published by Howman-Giles et al. [13]. HCT was used to calculate HCR. BClr was calculated using the method previously published by Ekman et al. [18]. An example can be seen in Figure 1. To calculate HEF, a deconvolved liver curve was created from the scans by drawing a region of interest (ROI) over the left ventricle of the heart (excluding the aorta) and a second ROI over the liver (excluding the gallbladder). Because ROI placement is critical for the accurate generation of the curves, the ROI must be placed over the parenchyma without inclusion of major bile ducts or the heart. Analysis is invalid if movement occurs. Therefore, planar motion correction was performed in the case of patient movement, using IntelliSpace Portal 11.1 (Philips Healthcare, Best, The Netherlands). Time–activity curves were generated using the heart curve as the input function and the liver curve as the output function for deconvolution analysis. Curves were interpolated to a uniform 1 s sampling time and filtered with a 120 s full width at half maximum Gaussian. Time zero for both curves was placed at the start of the increase in the heart ROI curve. The resulting deconvolved liver curve was a hypothetical measure of true liver function, representing the response to a direct ideal fast bolus injection into the hepatic artery.

HEF was then calculated as the ratio between the intercept of the fitted curve at time zero and the maximum value of the deconvolved liver curve, which was fitted between 7 and 30 min. In cases where the calculation produced an HEF value above 100%, these values were set to 100%, since an HEF above 100% is not possible clinically. HEF was estimated with the time range of 30 min for the retention phase (0–30 min).

The signal from the blood pool measured in the left ventricle of the heart was fitted with a mono-exponential function between 150 and 350 s after injection, yielding the blood clearance rate BClr in %/min according to Ekman et al. [18].

To calculate HCT, a mono-exponential function was fitted between 30 and 40 min of the liver ROI curve. HCT was defined as the time it takes for the activity to decrease by a factor of two [13]. Historically, HCT has been used as a measure of the excretion phase. However, for patients with very slow or negligible excretion, the calculated HCT can be very large and either positive or negative. This makes HCT unsuitable for statistical analysis, given that the calculated value for HCT can be misleadingly large, with no difference in the condition of the liver. We have used the HCR instead, which is equal to the relative (percentagewise) slope of the fitted mono-exponential curve. The following equation was used to calculate the HCR from HCT:

(1) $$HCR=\frac{ln2}{HCT}\cdot 100\%,$$

where HCT is measured in minutes and HCR in %/min.

HBSs were analysed by the nuclear medicine team. No excretion on HBS was defined as no excretion to the gastrointestinal tract after 24 h using visual interpretation.

### 2.4. Indocyanine Green Clearance

All ICG clearance measurements were performed at Copenhagen University Hospital—Rigshospitalet. All patients were fasting. ICG clearance was measured via two methods: a traditional blood sample or the LiMON pulse spectrophotometry device (Pulsion, Maquet Holding B.V. and Co., Rastatt, Germany). Because ICG-PDR is a more physically relevant and direct measure of elimination kinetics than ICG-R15 [19], ICG-PDR was chosen for further analyses. All ICG-plasma disappearance rate (PDR) values obtained via blood sample (ICG-PDRbs) were converted to ICG-PDRLi values, using the previously validated equation PDRbs = 0.83 × PDRLi for consistency [19].

For ICG clearance obtained via blood samples, two peripheral vein catheters were added in different veins. ICG (Verdye, Diagnostic Green, Aschheim-Dornach, Germany) was injected as a bolus of 0.25 mg/kg body weight of a 5 mg/mL solution in one of the catheters and flushed with 10 mL saline [20]. The amount of ICG remaining in the blood after injection decreases exponentially over approximately 20 min [21]. To calculate the ICG plasma disappearance rate (ICG-PDR, %/min) and ICG retention after 15 min (ICG-R15, %), blood samples were collected at the baseline and 5, 10, 15, and 20 min after ICG injection from the other catheter. Blood samples and a sample of the ICG standard were stored, protected from light. The plasma samples were centrifuged at 3000 rpm at room temperature, and the plasma and ICG samples were frozen at −18 °C for later analyses. The analyses for ICG clearance were performed using a spectrophotometer (ZeissPMQ-II, Carl Zeiss AG, Oberknocken, Germany).

For ICG clearance obtained via LiMON pulse spectrophotometry, one peripheral catheter was placed and the finger-clip used to measure ICG clearance was attached to the opposite hand. Primarily, the index finger was used for measurement, but for infants and younger children, either the thumb or combination of multiple fingers were used. A baseline measurement was obtained for one minute before ICG injection. A bolus of 0.25 mg/kg body weight of a 5 mg/mL solution was used in one of the catheters and flushed with 10 mL saline, similarly to the blood sampling technique. After injection, the LiMON probe performed pulse spectrophotometry for approximately 7.5 min, depending on the age and weight of the child, and calculated ICG-PDR and ICG-R15. Measurement failure due to patient or external factors resulted in no obtainable values of ICG-PDR and ICG-R15 in the display of the module.

ICG was prepared using 25 mg freeze-dried ICG, which were diluted with 5 mL sterile water, resulting in a 5 mg/mL ICG solution stored and kept dark and dry at room temperature for a maximum of 2 h before use.

### 2.5. Statistics

All data were entered into RedCap (14.5.17 Vanderbilt University, Nashville, TN, USA) and all analyses were conducted using STATA 18 (StataCorp, College Station, TX, USA). Disease groups, acute liver disease, LTx, cholestasis markers, portal blood flow, and the fibrosis degree were summarised as percentages (*n*). Age, blood tests, Fibroscan, and dynamic liver function tests were summarised as means (standard deviations (SD)) for normally distributed variables, or medians [interquartile ranges (IQR)] for non-normally distributed variables, based on a visual inspection of histograms and probability plots. Linear mixed effect models using patient ID as a random effect were used for the association of demographics, acute liver disease, LTx, disease groups, liver injury, synthetic function, cholestasis markers, portal blood flow, portal hypertension, and fibrosis degree with ICG-PDR, BClr, and HCR. For HEF, tobit mixed effects models were used with the upper limit of 100% to account for right censoring. Correlations between ICG-PDR, HCR, BClr, and HEF were assessed with Spearman’s ρ correlation coefficient for the first visit and linear mixed effect models for all visits, using patient ID as the random effect and ICG-PDR, BClr or HCR as outcome measurements. The models with ICG-PDR as the outcome also included total bilirubin as a fixed effect to account for the competitive inhibition between ICG and bilirubin. For HCR, BClr, and HEF, total bilirubin was only included as a fixed effect for bilirubin levels > 342 µM because mebrofenin only competes with total bilirubin above this level [22]. Children with missing data were included in the analyses with the available data. The rank sum and chi-squared tests were used to assess the difference in children with biliary atresia evaluated before and after Kasai.

## 3. Results

We included 131 children with a total of 200 visits. Of these, 56% had one visit, 36% had two visits, and 8% had three visits. The most common disease groups were biliary atresia and other liver diseases (Table 1). The median age at visit was 11.4 years [6.6; 15.6], 58% were female, and 31% had undergone LTx (Table 2). Summary statistics of liver disease markers can be seen in Table 2. ICG-PDR was measured in all children, HCR in 196 children, BClr in 184 children, and HEF in 179 children, as some did not have a cardiac input curve. The median ICG-PDR was 24.2%/min [17.0; 30.4], HCR was 3.3%/min [1.5; 4.6], BClr was 13.0%/min [8.7; 16.3], and HEF was 100% [87; 100]. ICG-PDR had the strongest correlation with HEF (ρ = 0.64, *p* < 0.001). The strongest correlation between HBS parameters was between HEF and HCR (ρ = 0.72, *p* < 0.001), whereas the weakest was seen between HCR and BClr (ρ = 0.39, *p* < 0.001) (Table 3). No adverse events were recorded in our study.

### 3.1. Disease Markers in Children with Liver Transplantation and Differences in Biliary Atresia Children with and Without Kasai

Liver blood markers in children with LTx were mostly within the normal levels and only a few of the children had cholestasis (Table 4). The degree of fibrosis varied more, but 87% had fibrosis at a degree of two or less. Of the children with biliary atresia, seven were evaluated before Kasai. No difference was seen for sex (*p* = 0.56) and INR (1.1 [1.0; 1.2] vs. 1.2 [1.1; 1.3], *p* = 0.057), but higher values were seen for ALT (108 U/L [85; 192] vs. 36 U/L [26; 55], *p* < 0.001), GGT (325 U/L [300; 414] vs. 27 U/L [15; 98], *p* < 0.001), and total bilirubin (131 µM [83; 148] vs. 8 µM [5; 15], *p* < 0.001).

### 3.2. Markers of Liver Disease and Their Association with Dynamic Liver Function Tests

Acute liver disease and LTx were associated with select dynamic liver function tests, while no significant differences were observed across disease groups (Table 5). Markers of liver injury, synthetic dysfunction, and cholestasis—such as elevated ALT, increased INR, decreased prothrombin time (PP), and reduced albumin—were linked to diminished liver function, as shown in Table 5. Cholestasis markers, including absent HBS excretion, total bilirubin ≥ 20 μM, and elevated GGT, also correlated with poorer liver function. Liver cirrhosis was associated with decreased function across all measures. Additionally, Fibroscan measurements indicating portal hypertension correlated with reduced liver function on both ICG-PDR and HBS markers, whereas low platelet counts were only associated with ICG-PDR and BClr. Lastly, a portal blood flow below 20 cm/s was linked to decreased ICG-PDR, with no significant association with HBS parameters (Table 5).

### 3.3. BClr in Children with Normal HEF

Of the 179 HEF measurements, 130 had HEF > 90% with a median BClr of 14.4%/min [11.9; 17.2]. Those with HEF ≤ 90% had a lower median BClr of 7.1%/min [4.5; 10.7] (*p* < 0.001). There was no correlation between ICG-PDR and BClr (ρ = 0.24, *p* = 0.028) in those with HEF > 90%, whereas in children with HEF ≤ 90%, a correlation was seen (ρ = 0.67, *p* < 0.001). Of the 130 with HEF > 90%, 16 had an ICG-PDR < 18.5%/min and BClr ranging from 4.5 to 19.4. In patients with HEF > 90% and total bilirubin ≥ 20 µM, BClr was 3.5%/min (95% CI 0.6; 6.4) lower than in those with total bilirubin < 20 µM. BClr also increased 4.8%/min (95% CI 1.4; 8.4) for each percentage increase in platelets. None of the other liver disease parameters were associated with BClr in those with HEF > 90%.

## 4. Discussion

In this large paediatric study, we found that ICG clearance and the quantitative parameters of HBS were correlated, with the strongest correlation between ICG-PDR and HEF and the weakest between ICG-PDR and BClr. Additionally, ICG clearance and quantitative parameters of HBS were associated with most of the conventional liver function markers, i.e., liver injury, reduced synthetic function, cholestasis, cirrhosis, and portal hypertension, whereas only ICG clearance was associated with portal blood flow.

### 4.1. Correlation Between ICG-PDR and HBS Parameters

We found that ICG-PDR correlated with all HBS parameters, and the strongest correlation was between ICG-PDR and HEF. Few studies have previously evaluated the correlation between ICG clearance and HBS, and only in adults. These studies found a similar or stronger correlation than in our study. Wang et al. found a correlation of ρ = 0.80 between HEF and the ICG clearance rate and Erdogan et al. found a correlation of ρ = 0.73 between the HBS liver uptake rate and the ICG 15 min clearance rate [11,12]. The slight difference between Wang et al. and our results may be caused by different factors. Firstly, the adult studies were done on patients with liver tumours before resection. There is a distinction between tumours occurring in an otherwise healthy liver and diseases that affect the entire liver, as seen in most participants in our study. Liver disease-related factors may play a role (e.g., bilirubin influence ICG clearance measurement, but only HBS at very high levels). Secondly, younger children often find it challenging to remain still for the full 60 min of the HBS scan, even with distractions. Although technical difficulties can occur, they rarely affect visual interpretation. ROI placement is critical for accurate curve generation—particularly correct positioning over the parenchyma and the exclusion of major bile ducts and the heart—but it is more difficult in younger children because of their proximity. Planar motion correction was consistently applied to compensate for movement during acquisition, and appropriate immobilisation was ensured throughout the procedure. Thirdly, the ICG doses differed between studies. We administered 0.25 mg/kg, whereas the adult studies gave 0.5 mg/kg or 25 mg, regardless of weight [11,12]. ICG-PDR performed with 0.25 mg/kg are highly correlated with ICG-PDR performed with 0.50 mg/kg (ρ = 0.95), but with a trend towards lower percentage differences between dosages with higher ICG-PDR [20]. This suggests that dosage is more important in children with reduced liver function.

BClr and ICG-PDR both measure blood clearance and we hypothesised them to be the most strongly correlated measures; however, the observed correlation was only ρ = 0.39. When looking at children with low HEF, the correlation between ICG-PDR and BClr is high, whereas it was low in those with normal HEF. This may be due to high variance for both ICG-PDR and BClr in those with high HEF. These findings suggest that when the liver is functioning well enough to have a normal HEF, other parameters might not provide additional information.

### 4.2. Dynamic Liver Function Tests and Markers of Liver Disease

We found that reduced ICG-PDR and HBS parameters were associated with markers of liver injury, reduced synthetic function, cholestasis, cirrhosis, and portal hypertension. This is in line with previous studies where more severe liver disease, measured with conventional liver markers or disease severity classification, is associated with reduced ICG clearance and HBS [9,10,12,14,23,24]. Our findings suggest that ICG clearance and HBS are useful in evaluating liver function, as they are associated with most of the conventional markers, and they can individually provide information that is normally assessed with multiple markers.

Reduction in the portal blood flow was only associated with ICG-PDR. The association with ICG-PDR is expected due to the kinetics of ICG-PDR [10]. For HBS, HEF and HCR are measures which can be normal even with reduced portal flow, but BClr is expected to be dependent on portal blood flow. The lack of association may be because portal blood flow is a category variable and BClr is not influenced before the portal blood flow is further reduced.

We found no differences in ICG-PDR and HBS parameters for any of the disease groups. Previous studies have found that those with cholestasis without parenchymal disease had higher HEF than those with parenchymal disease [13,23]. Most of the children in our cohort had affected parenchyma, regardless of the disease aetiology, which can explain the lack of difference in liver function test between disease groups. LTx was not associated with ICG clearance, but it was associated with quantitative parameters of HBS. This is most likely due to the change in anatomy and the subsequent drawing of ROI in HBS, which is not an issue in ICG clearance. The change in biliary and intestinal anatomy can make it difficult to make sure the right structures are excluded. This is consistent with prior research demonstrating inconsistent findings regarding the utility of HBS after LTx. For example, Gençoğlu et al. reported no correlation between conventional liver function markers and HEF, whereas Zou et al. observed a significant association between HEF and conventional liver markers following transplantation [25,26]. Both studies performed HBS within a month of transplantation, whereas the time varies in our study and includes HBSs performed years later. This could have influenced our findings, as the new liver will get fibrotic over time [27].

### 4.3. Clinical Relevance

The findings in our study suggest that ICG and HBS could both be useful in the evaluation of disease progression. In the clinical management of the patient, it is important to make the assessment of liver disease status as precise, cost-effective, and easy for the patients as possible. ICG and HBS are both associated with cirrhosis, as well as other markers of liver disease. However, ICG is fast and minimally invasive, and can be conducted in the outpatient clinic by trained nurses, whereas HBS gives additional information on local liver function and excretion, but it is more extensive and requires more expertise. If the ICG clearance suggests worsening liver function, HBS can be used to give more information on the anatomical location and the excretion. Following further longitudinal studies, these dynamic liver function tests can hopefully lead to an easier way of assessing liver fibrosis and the function of the non-fibrotic tissue, reducing the need for liver biopsies. Liver biopsies in children require general anaesthesia and hospitalisation, as they carry the risk of infection and bleeding.

### 4.4. Challenges

**Figure 2:**
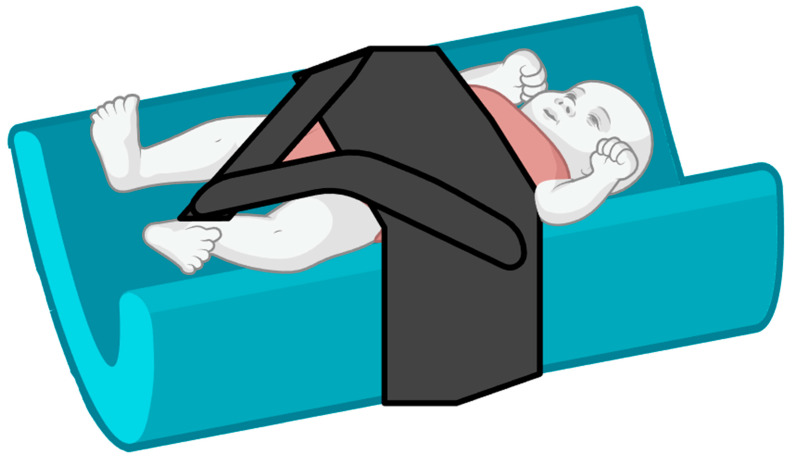
An example of fixation for hepatobiliary scintigraphy to reduce movement. Created in BioRender. Brix, V. (2026) https://BioRender.com/ivpfe3b.

For some scans, HEF could not be determined due to the missing signal or ROI of the heart curve, low quality of the heart curve, or low quality of the liver curve. Most commonly, it was because the heart was not in the scanned field of view or due to patient movement, which could not be corrected by motion correction software (IntelliSpace Portal 11.1, Philips Healthcare, Best, The Netherlands). It is therefore of the utmost importance to ensure that the heart is within the field of view when scanning the patient. This can be achieved using a radioactive source pen, which is standard equipment at any nuclear medicine department. Although this step was included in our study’s procedural guidelines, it should be further emphasised. Patient movement can be minimised using distraction techniques and appropriate immobilisation (Figure 2). Additionally, it is important to exclude the signal from the bile tree for proper calculation. This can be challenging as, for example, children with biliary atresia or LTx have altered anatomy.

### 4.5. Limitations

This study has some limitations. Firstly, while most of the ICG-PDR values were obtained via LiMON spectrophotometry, 16% were obtained via blood sample and converted to LiMON values using the previously validated equation PDRbs = 0.83∙PDRLi [14]. This was due to initial problems obtaining LiMON values at the beginning of the study (due to small finger size, etc.). Secondly, the injection technique was found to be important during the study, as the deconvolution analysis technique requires a fast intact bolus. Upper limb injections, preferably in the antecubital fossa, were performed. Thirdly, the children were required to remain still for 60 min, which was difficult when fasting and young. Many children with liver disease also have neurocognitive problems, creating addition challenges. Also, this difference in ability in cooperation, depending both on age and disease severity, can cause differential bias in the cohort, making it harder to properly place ROI in younger/more ill patients. To address this, motion correction was performed as previously mentioned. Fourthly, if the correct zoom had not been used, it was more difficult to place the ROIs correctly. In those cases, the ROIs have been evaluated by a second person to ensure valid results and were redrawn if necessary. Fifthly, the study was only conducted in one centre, which could reduce generalisability. However, both ICG clearance and HBS were conducted using manufacture standards and standard protocols and both are performed by specially trained personnel. All of this improves generalisability. Lastly, HEF was censored at 100% in more than half the children. To address this, tobit regression was used. Tobit regression is a censored regression model that explicitly models the probability of being censored and the relationship to the uncensored data. It is a robust model, as it takes the censored data into account instead of discarding them, allowing for a robust conclusion, even with censoring.

## 5. Conclusions

ICG clearance and HBS parameters are correlated, with the strongest correlation between ICG-PDR and HEF. ICG clearance and HBS parameters are also associated with most conventional liver function markers of liver injury, reduced synthetic function, cholestasis, cirrhosis, and portal hypertension. This suggests the usefulness of the ICG clearance and HBS parameters in the evaluation of liver function. Further studies to evaluate the predictive value of ICG clearance and HBS parameters in disease progression are needed to further determine their role in the clinic.

## Figures and Tables

**Table 1:** Diseases.

Disease Groups	Patients (*n* = 131) % (*n*)	Visits (*n* = 200) % (*n*)	Percentage of Visits with LTx % (*n*)
**Metabolic/genetic**	**19% (25)**	**16% (32)**	**38% (12)**
Alagille syndrome	5% (7)	5% (9)	
Alpha-1 antitrypsin deficiency	4% (5)	3% (5)	
Carbamoyl phosphate synthetase 1 deficiency	1% (1)	0.5% (1)	
Citrullinaemia	1% (1)	1% (2)	
Cystic fibrosis	1% (1)	1% (2)	
Galactosaemia	1% (1)	0.5% (1)	
Tyrosinaemia	1% (1)	0.5% (1)	
Progressive familial intrahepatic cholestasis	1% (1)	0.5% (1)	
Polycystic kidney disease	2% (3)	3% (6)	
Wilson’s disease	3% (4)	2% (4)	
**Biliary atresia**	**26% (34)**	**31% (62)**	**55% (34)**
**Autoimmune hepatitis**	**21% (28)**	**22% (45)**	**2% (1)**
**Other**	**34% (44)**	**31% (61)**	**25% (15)**
Infectious hepatitis (B, C, Epstein–Barr)	8% (11)	8% (16)	
Metabolic dysfunction–associated steatotic liver disease	2% (3)	3% (5)	
Toxic (mushroom, oral contraception, and paracetamol)	5% (7)	4% (8)	
Choledochal cyst	2% (2)	1% (2)	
Biliary duct obstruction	1% (1)	0.5% (1)	
Hepatoblastoma	2% (3)	3% (6)	
Rhabdomyosarcoma	1% (1)	0.5% (1)	
Other cancers	1% (1)	2% (3)	
Portal vein thrombosis	2% (3)	2% (3)	
Enterocolitis	1% (1)	1% (2)	
Immaturity	1% (1)	0.5% (1)	
Cryptogenic cholestasis	1% (1)	0.5% (1)	
Cryptogenic acute liver failure	2% (2)	2% (3)	
Cryptogenic cirrhosis	3% (4)	3% (5)	
Unknown liver disease	2% (3)	2% (4)	

Bold text represent main categories.

**Table 2:** Demographics and liver parameters are based on the 200 visits, not the 131 patients.

	Visits, *n*	% (*n*) or Median [IQR] for Visits
Age (years)	200	11.4 [6.6; 15.6]
Sex, female	200	58% (116)
Acute liver disease	200	12% (23)
Liver transplanted	200	31% (62)
**Blood test**		
ALT (U/L)	197	42 [22; 108]
GGT (U/L)	194	33 [16; 106]
Total bilirubin (µM)	197	9 [6; 17]
Conjugated bilirubin (µM)	60	8 [4; 62]
INR	200	1.1 [1.0; 1.2]
PP	199	0.72 [0.60; 0.87]
Platelets (10^9^/L)	200	231 [148; 305]
Albumin (g/L)	197	37 [34; 40]
**Cholestasis**		
No excretion on hepatobiliary scintigraphy	200	7% (13)
Total bilirubin ≥ 20 µM	197	22% (44)
**Liver blood flow**		
Portal blood flow < 20 cm/s	199	21% (41)
**Liver fibrosis**		
Fibroscan (kPa)	182	8.0 [4.8; 14.9]
Biopsy (Metavir)	160	
No fibrosis		26% (42)
Mild fibrosis		21% (33)
Significant fibrosis		28% (44)
Severe fibrosis		8% (12)
Cirrhosis		18% (29)

Abbreviations: ALT = Alanine transaminase, GGT = gamma-glutamyl transferase, INR = international normalised ratio; PP = prothrombin–proconvertin clotting time.

**Table 3:** Correlation between dynamic liver function test ICG-PDR, HEF, BClr, and HCR.

	ICG Plasma Disappearance Rate (%/min)		Hepatic Clearance Rate (%/min)		Blood Clearance Rate (%/min)	
**Spearman’s coefficients**	**rho**	***p*** **-Value**	**Rho**	***p*** **-Value**	**rho**	***p*** **-Value**
Hepatic extraction fraction (%)	0.64	<0.001	0.72	<0.001	0.64	<0.001
Blood clearance rate (%/min)	0.56	<0.001	0.39	<0.001	-	-
Hepatic clearance rate (%/min)	0.52	<0.001	-	-	-	-
**Linear mixed effects model**	***β*** **(95% CI)**	***p*** **-Value**	***β*** **(95% CI)**	***p*** **-Value**	***β*** **(95% CI)**	***p*** **-Value**
Hepatic extraction fraction (%)	0.2 (0.1; 0.2)	<0.001	0.05 (0.04; 0.06)	<0.001	0.1 (0.1; 0.1)	<0.001
Blood clearance rate (%/min)	0.5 (0.2; 0.8)	<0.001	0.13 (0.07; 0.18)	<0.001	-	-
Hepatic clearance rate (%/min)	1.9 (1.3; 2.5)	<0.001	-	-	-	-

BClr = blood clearance rate, HEF = hepatic extraction fraction, HCR = hepatic clearance rate, and ICG = indocyanine green. Linear mixed effect models with ID as a random effect were used with the ICG plasma disappearance rate, blood clearance rate and hepatic clearance rate as an outcome measurement. The models with ICG-PDR as the outcome also included total bilirubin as a fixed effect to account for the competitive inhibition between ICG and bilirubin. For HCR, BClr, and HEF, total bilirubin was only included as a fixed effect for bilirubin levels > 342 µM, as mebrofenin only competes with total bilirubin above this level. Spearman’s rho correlation coefficient was used.

**Table 4:** Liver parameters in patients with liver transplantation.

	Visits, *n*	% (*n*) or Median [IQR] for Visits
**Blood test**		
ALT (U/L)	62	32.5 [26; 49]
GGT (U/L)	61	27 [15; 73]
Total bilirubin (µM)	62	7.5 [5; 12]
INR	62	1.2 [1.1; 1.2]
**Cholestasis**		
No excretion on hepatobiliary scintigraphy	62	2% (1)
Total bilirubin ≥ 20 µM	62	6% (4)
**Liver fibrosis**		
Fibroscan (kPa)	60	8.4 [5.5; 17.5]
Biopsy (Metavir)	60	
No fibrosis		33% (20)
Mild fibrosis		25% (15)
Significant fibrosis		28% (17)
Severe fibrosis		7% (4)
Cirrhosis		7% (4)

Abbreviations: ALT = Alanine transaminase, GGT = gamma-glutamyl transferase, and INR = international normalised ratio.

**Table 5:** Dynamic liver function tests in relation to demographic and clinical variables.

	ICG Plasma Disappearance Rate (%/min)		Hepatic Extraction Fraction (%)		Blood Clearance Rate (%/min)		Hepatic Clearance Rate (%/min)	
	*β* (95% CI)	*p*-Value	*β* (95% CI)	*p*-Value	*β* (95% CI)	*p*-Value	*β* (95% CI)	*p*-Value
Age (years)	0.06 (−0.2; 0.3)	0.64	4.3 (2.4; 6.2)	<0.001	0.06 (−0.09; 0.21)	0.40	0.09 (0.03; 0.14)	0.003
Sex, female	−0.1 (−2.9; 2.7)	0.94	−14.7 (−36.8; 7.4)	0.19	−1.15 (−2.79; 0.47)	0.16	0.10 (−0.54; 0.74)	0.75
Acute liver disease	−3.1 (−7.4; 1.2)	0.16	−28.3 (−59.6; −3.0)	0.077	−3.47 (−5.86; −1.07)	0.005	−0.90 (−1.83; 0.02)	0.055
Liver transplanted	1.0 (−1.9; 4.0)	0.49	47.4 (21.6; 73.1)	<0.001	2.47 (0.78; 4.15)	0.004	1.79 (1.18; 2.39)	<0.001
**Disease groups**								
Biliary atresia	Ref.		Ref.		Ref.		Ref.	
Metabolic/genetic	3.7 (−0.4; 7.9)	0.081	−4.6 (−38.2; 28.9)	0.79	1.80 (−0.73; 4.32)	0.16	−0.51 (−1.46; 0.44)	0.30
Autoimmune hepatitis	2.6 (−1.3; 6.4)	0.19	16.0 (−15.1; 47.0)	0.31	1.03 (−1.19; 3.26)	0.36	−0.19 (−1.06; 0.69)	0.67
Other	2.9 (−0.6; 6.3)	0.11	12.1 (−16.7; 40.8)	0.41	1.82 (−0.24; 3.89)	0.084	0.35 (−0.45; 1.14)	0.39
**Liver injury**								
Log10 ALT	−3.8 (−6.1; −1.6)	0.001	−49.9 (−66.5; −33.3)	<0.001	−2.53 (−3.82; −1.24)	<0.001	−1.30 (−1.77; −0.83)	<0.001
**Synthetic function**								
INR	−7.7 (−14.0; −1.4)	0.016	−84.7 (−129.1; −40.3)	<0.001	−8.17 (−11.4; −4.93)	<0.001	−1.72 (−3.02; −0.42)	0.010
PP	11.1 (4.5; 17.7)	0.001	74.3 (20.9; 127.8)	0.006	7.41 (3.75; 11.1)	<0.001	1.46 (−0.01; 2.93)	0.051
Albumin (g/L)	0.7 (0.5; 1.0)	<0.001	5.4 (3.6; 7.3)	<0.001	0.42 (0.28; 0.57)	<0.001	0.17 (0.12; 0.23)	<0.001
**Cholestasis**								
No excretion on hepatobiliary scintigraphy	−10.1 (−16.6; −3.5)	0.003	−99.4 (−134.3; −64.5)	<0.001	−6.54 (−9.72; −3.37)	<0.001	−3.52 (−4.64; −2.41)	<0.001
Total bilirubin ≥ 20 µM	−10.6 (−13.8; −7.4)	<0.001	−77.3 (−96.0; −58.5)	<0.001	−6.33 (−7.92; −4.73)	<0.001	−2.67 (−3.26; −2.08)	<0.001
Log10 GGT	−5.5 (−7.9; −3.0)	<0.001	−56.1 (−75.8; −36.3)	<0.001	−2.46 (−3.95; −0.97)	0.001	−1.79 (−2.31; −1.26)	<0.001
**Portal blood flow**								
Portal blood flow < 20 cm/s	−6.1 (−9.3; −3.0)	<0.001	−24.0 (−49.0; 1.0)	0.060	−1.36 (−3.29; 0.56)	0.17	−0.63 (−1.34; 0.08)	0.084
**Portal hypertension**								
Log10 Platelets	10.2 (5.0; 15.4)	<0.001	29.6 (−11.4; 70.6)	0.16	4.27 (1.18; 7.35)	0.007	0.57 (−0.65; 1.78)	0.36
Fibroscan (kPa)	−0.1 (−0.2; −0.02)	0.013	−1.0 (−1.7; −0.3)	0.003	−0.06 (−0.12; −0.01)	0.013	−0.03 (−0.05; −0.01)	0.004
**Liver fibrosis on biopsy (Metavir** )								
No fibrosis	Ref.		Ref.		Ref.		Ref.	
Mild fibrosis	−4.8 (−8.9; −0.7)	0.022	−21.2 (−55.7; 13.3)	0.23	−0.92 (−3.31; 1.47)	0.45	−1.17 (−2.12; −0.23)	0.015
Significant fibrosis	−3.5 (−7.4; 0.3)	0.072	−3.9 (−37.2; 29.4)	0.82	−0.53 (−2.75; 1.70)	0.64	−0.72 (−1.60; 0.16)	0.11
Severe fibrosis	−5.0 (−10.8; 0.9)	0.096	−21.1 (−67.1; 24.9)	0.37	1.61 (−1.73; 4.96)	0.35	−1.62 (−2.97; 0.26)	0.020
Cirrhosis	−10.1 (−14.6; −5.5)	<0.001	−63.6 (−99.0; −28.3)	<0.001	−4.52 (−7.05; −1.98)	<0.001	−2.61 (−3.61; −1.61)	<0.001

Abbreviations: ALT = Alanine transaminase, GGT = gamma-glutamyl transferase, INR = international normalised ratio; PP = prothrombin–proconvertin clotting time, and ICG = indocyanine green. Linear mixed effect models with ID as a random effect were used for the ICG plasma disappearance rate and hepatic clearance rate. The models with ICG-plasma disappearance rate as the outcome also included total bilirubin as a fixed effect to account for the competitive inhibition between ICG and bilirubin. For HCR and HEF, total bilirubin was only included as a fixed effect for bilirubin levels > 342 µM, as mebrofenin only competes with total bilirubin above this level. Tobit mixed effects models with 100% as right censoring were used for the hepatic extraction fraction.

## Data Availability

According to the Danish Act on Data Protection, personal data cannot be shared with others without prior approval from the Danish Data Protection Agency. The data presented in this study are available upon request from the corresponding author, due to legal restrictions.

## Abbreviations

The following abbreviations are used in this manuscript:

HBS	hepatobiliary scintigraphy
ICG	indocyanine green
LTx	liver transplantation
PLD	paediatric liver disease
[^99m^Tc]-IDA	99mTc-labelled analogue of iminodiacetic acid
ALT	alanine aminotransferase
GGT	gamma-glutamyl transferase
INR	international normalised ratio
PP	prothrombin–proconvertin clotting time
HEF	hepatic extraction fraction
BClr	blood clearance
HCR	hepatic clearance rate
HCT	hepatic clearance time
ROI	region of interest
PDR	plasma disappearance rate
ICG-PDR	ICG plasma disappearance rate
ICG-R15	ICG retention after 15 min
IQR	interquartile range
SD	standard deviation
